# Factors Associated with Early Discharge after Thoracoscopic Lobectomy: Results from the Italian VATS Group Registry

**DOI:** 10.3390/jcm11247356

**Published:** 2022-12-11

**Authors:** Pio Maniscalco, Nicola Tamburini, Nicolò Fabbri, Francesco Quarantotto, Giovanna Rizzardi, Dario Amore, Camillo Lopez, Roberto Crisci, Lorenzo Spaggiari, Giorgia Valpiani, Luca Bertolaccini, Giorgio Cavallesco

**Affiliations:** 1 Department of General Thoracic Surgery, Sant’Anna University Hospital, 44124 Ferrara, Italy; 2 Department of General Surgery, Sant’Anna University Hospital, 44124 Ferrara, Italy; 3 Department of Thoracic Surgery, Cliniche Humanitas Gavazzeni, 24125 Bergamo, Italy; 4 Department of Thoracic Surgery, Monaldi Hospital, 80131 Naples, Italy; 5 Department of Thoracic Surgery, Vito Fazzi Hospital, 73100 Lecce, Italy; 6 Department of Thoracic Surgery, University of L’Aquila, 64100 L’Aquila, Italy; 7 Department of Thoracic Surgery, IEO, European Institute of Oncology IRCCS, 20141 Milan, Italy; 8 Department of Oncology and Hemato-Oncology, University of Milan, 20141 Milan, Italy; 9 Research Innovation Quality and Accreditation Unit, Sant’Anna University Hospital, 44124 Ferrara, Italy

**Keywords:** thoracoscopic lobectomy, hospitalization, length of hospital stay, lung cancer, risk factors

## Abstract

Objective. There are limited data for estimating the risk of early discharge following thoracoscopic lobectomy. The objective was to identify the factors associated with a short length of stay and verify the influence of these variables in uncomplicated patients. Methods. We reviewed all lobectomies reported to the Italian VATS Group between January 2014 and January 2020. Patients and perioperative characteristics were divided into two subgroups based on whether or not they met the target duration of stay (≤ or >4 days). The association between preoperative and intraoperative variables and postoperative length of stay (LOS) ≤4 days was assessed using a stepwise multivariable logistic regression analysis to identify factors independently associated with LOS and factors related to LOS in uncomplicated cases. Results. Among 10,240 cases who underwent thoracoscopic lobectomy, 37.6% had a hospital stay ≤4 days. Variables associated with LOS included age, hospital surgical volume, Diffusion Lung CO % (81 [69–94] vs. 85 [73–98]), Forced Expiratory Volume (FEV1) % (92 [79–106] vs. 96 [82–109]), operative time (180 [141–230] vs. 160 [125–195]), uniportal approach (571 [9%] vs. 713 [18.5%]), bioenergy sealer use, and pain control through intercostal block or opioids (*p* < 0.001). Except for FEV1 and blood loss, all other factors emerged significantly associated with LOS when the analysis was limited to uncomplicated patients. Conclusions. Demographic, clinical, and surgical variables are associated with early discharge after thoracoscopic lobectomy. This study indicates that these characteristics are associated with early discharge. This result can be used in association with clinical judgment to identify appropriate patients for fast-track protocols.

## 1. Introduction

Reducing hospital length of stay is a desirable method to cut costs and enhance outcomes in case of limited healthcare resources. The debate over healthcare costs is still an important topic, and many researchers have attempted to influence healthcare costs following surgical procedures by reducing hospital length of stay [1,2,3]. Thoracic surgery, especially lung cancer surgery, is not an exception [4,5]. Although the use of advanced minimally invasive techniques for lung resection has dramatically reduced mortality, hastened recovery, and reduced the duration of hospitalization [6], thoracic surgeons continue to face ever-increasing pressure to discharge their patients earlier in order to control costs. Perioperative care programs in elective lung surgery based on Enhanced Recovery After Surgery (ERAS) Society recommendations have been increasingly described to reduce the length of hospital stay (LOS) and lower the postoperative complication and readmission [7,8,9]. Although there are several studies on the factors that contribute to discharge after chest surgery, the majority of them focus on cardiac surgical patients. Furthermore, many studies attempting to develop fast-track protocols after VATS lobectomy analyzed a limited number of patients and have not yet identified recovery and length of stay predictors. For this reason, using objective measurements to guide decision-making may help to facilitate early discharge.

In this report, we used the data from the Italian VATS Registry to identify factors influencing recovery and length of stay after VATS lobectomy for NSCLC that would be appropriate for fast-tracking. We hypothesized that hospital length of stay could be predicted by some factors known before the operation but also influenced by other factors not known before surgery.

## 2. Materials and Methods

### 2.1. Ethical Statement

The VATS group registry received in 2014 the Institutional Review Board approval (No. 81/2014/O/Oss). The hospital ethics committee approved this study (No. 171173). The data were anonymously achieved according to the International Conference on Harmonization Guidelines for Good Clinical Practice [10].

### 2.2. Data Source

The Italian VATS Group Registry, recently updated to the 2.0 registry, provided all these research data. In January 2014, this retrospective database was established to collect data on VATS lobectomies performed by 58 Italian-certified thoracic surgical institutions. The VATS Group Database is a validated, risk-adjusted, and outcomes-based source, gathering data in standardized forms for the following variables: patient demographics, surgical procedures, medical history, cancer stage, and outcome. The VATS Group Database implements rigorous quality assurance and safety procedures to maintain data accuracy and security. The current analysis was reviewed and approved for scientific merit and feasibility by the VATS Group Scientific Committee, and the preliminary results were presented at the annual VATS Group meeting. The manuscript was written according to the STROCSS statement [11]. A STROCSS checklist can be found in the Supplementary Material.

### 2.3. Patient Population and Study Design

We performed a retrospective study of patients who underwent VATS lobectomy as the primary procedure between January 2014 and January 2020 using a national prospectively maintained database. Patients who died were excluded from the study. In order to analyze the factors related to the length of stay, the study design provides a comparison between two groups based on the LOS distribution. Therefore, patients were grouped into two groups using second tertiles of LOS distribution (4 days) as a cut-point. The first tertile group, identified as “LOS ≤ 4”, included all patients discharged within four days after surgery. The second group, called “LOS > 4”, included all cases discharged after the fourth postoperative day. In the absence of a prescribed definition of LOS in the literature and to confirm the appropriateness of our selection, we consulted the surgeons of the VATS registry for their judgement, who agreed on this cut-off value. Moreover, the use of the LOS at the second tertiles is consistent with other studies.

### 2.4. Covariates

We compared selected clinical variables to analyze the presence of a possible association with length of stay: data were divided into preoperative, intraoperative, and postoperative. Furthermore, three groups, obtained from tertiles distribution, were created according to the number of cases of the different centers and considered as a significant variable: group A (less than 200 cases), group B (number of cases between 201 and 364), and group C (above 364 patients).

### 2.5. Statistical Analysis

The Shapiro–Wilk test was used to test for the normality of the distribution of the continuous variables. In the presence of symmetry of the distributions, the variables are represented with mean and standard deviation (SD) or, in the case of non-normal distribution, with the median value and interquartile range [1Q–3Q]; categorical data are expressed as total numbers and percentages. Student’s *t*-test was used in the normality of data; the non-parametric Mann–Whitney test was used for the non-normally distributed variables, and the chi-squared test was used for categorical data to compare groups.

We estimated unadjusted and adjusted logistic regression models to analyze the effects of the independent variables on hospital stay based on the presence or absence of complications. We simplified multiple models with a stepwise backward procedure to obtain a more basic model containing only the significant factors. A significance level of 0.2 was required to be considered in the model, and a level of 0.05 was required not to be dropped. Adjusted Odds Ratios (ORs) and 95% Confidence Intervals (95% CIs) were presented. All *p*-values are two-sided, with statistical significance evaluated at the 0.05 α level. All tests were performed with Stata 13.0 statistical software (Stata Corp., College Station, TX, USA).

## 3. Results

During the research period, a total of 10,240 cases were included in the analysis. Of these, 968 (9.5%) were finally converted to thoracotomy; thus, they were included in the study with the intention to treat analysis. The LOS ≤ 4 groups accounted for 37.6% (*n* = 3857), with 54.9% of male patients and 45.1% of female patients with a median age of 68 [61–73). The LOS > 4 group included 62.4% (*n* = 6383) of the cases with 4002 male patients (62.7%) and 2381 females (37.3%), with a median age of 70 [63–75] years. There was a substantial predominance of males and an older mean older age in the LOS > 4 groups (Table 1).

The patient’s preoperative characteristics are reported in Table 2 and Table 3. The percentage of patients presenting a history of myocardial infarction (*p* < 0.001), congestive heart failure (*p* < 0.001), and peripheral and cerebrovascular illness (*p* < 0.001) was significantly greater in the LOS > 4 groups than in the LOS ≤ 4 group. The final Charlson comorbidity index score demonstrated significantly lower values of 4 [3–5] vs. 5 [3–6] in LOS ≤ 4 groups (*p* < 0.001). Furthermore, in the LOS >4 groups, the incidence of COPD was higher (23.7% vs. 16.5%, *p* < 0.001), and the median values of diffusing capacity for carbon monoxide (DLCO) corrected for alveolar volume (VA) values were lower 81 [69–94] vs. 85 [73–98] (*p* < 0.001). Same results were obtained for the analysis of Fev1% values 92 [79–106] vs. 96 [82–109] (*p* < 0.001).

**Figure 1:**
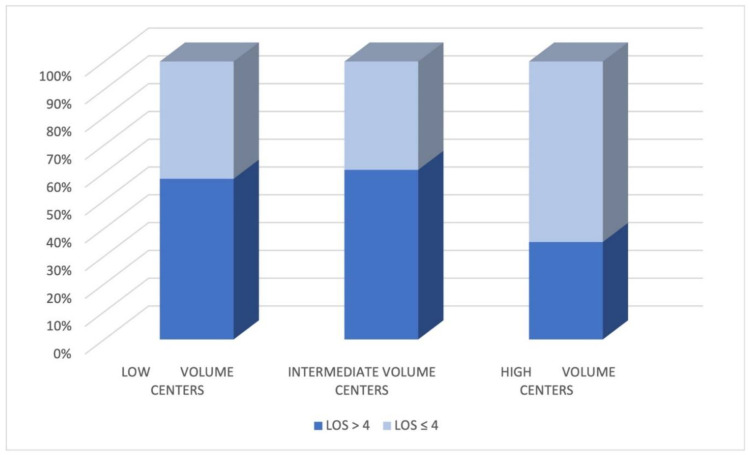
Correlations between length of hospital stay (LOS) and surgical centers’ activities volumes.

The association between intraoperative features with outcomes is shown in Table 4 and Table 5. The duration of surgery was significantly lower in the LOS ≤ 4 groups (160 [125–195] vs. 180 [141–230] minutes, *p* < 0.001) as also the intraoperative blood loss (*p* < 0.001). Cases that did not require conversion showed shorter hospital stays (*p* < 0.001). Furthermore, the uniportal approach, opioids infusion, absence of pleural adhesions, and middle lobectomies were associated with shorter LOS. The analysis of the relationship between the hospital VATS lobectomy volume and length of stay revealed a significant association between high-volume centers and dismission (Figure 1). It is essential to highlight that in the low and intermediate volume centers, the LOS > 4 group number is more prominent than LOS < 4, while in high volume centers, the two groups are comparable. Postoperative data are summarized in Table 6. In the LOS ≤ 4 group, 92.9% of patients did not experience postoperative complications [12], and 7.1% of patients had at least one complication, while in the LOS > 4 group, this percentage was significantly higher (38.8%, *p*-value < 0.001).

The univariable linear regressions in Table 7 showed that conversion was significantly associated with a longer length of hospital stay. Table 8 shows the variables that remained statistically significant in the multiple logistic regression analysis. An age < 70 years, the absence of COPD diagnosis, operative time < 240 min, lobectomy hospital volume, FEV1 and DLCO/VA < 70%, intraoperative blood loss < 600 mL, uniportal approach, bioenergy sealer use, conversion, and pain control through intercostal block or opioids were factors associated with LOS. Furthermore, to consider the confounding effect of complications, the regression analysis focused on the effects of the independent variables on hospital stay of more than or less than 4 days based on the presence or absence of complications (Table 6). Except for FEV1 and blood loss, all the other determinants remained significantly associated with LOS ≤ 4 days.

## 4. Discussion

The length of hospital stay after surgery is a significant patient-centered outcome essential to patients, clinicians, and payers [13]. This research revealed different demographic and perioperative characteristics associated with LOS, which have therapeutic relevance in accelerating postoperative recovery and expediting discharge. We found that patients with age > 70 years and diagnosed with COPD with FEV1 and DLCO < 70% are less likely to have early discharge. Additionally, patients undergoing lobectomy in high hospital volume with operative time < 240 min, intraoperative blood loss < 600 mL, uniportal approach, bioenergy sealer use, conversion, and pain control through an intercostal block or opioids surgery were more likely to have early discharge. Therefore, these factors can predict which patients would be most eligible for early discharge after VATS lobectomy. LOS has previously been investigated as a quality metric for pulmonary lobectomy. Wright [4] identified several patient factors associated with prolonged hospital stay (PLOS), including age, male gender, Zubrod score, and various comorbidities. However, their model considered prolonged LOS a surrogate for surgical morbidity. Indeed, patients with PLOS had significantly more postoperative adverse events than patients without PLOS. Similar conclusions were reported by other authors [5]. Our findings support the published research since complications were associated with longer LOS: 91.1% of cases with postoperative complications had a hospital stay longer than 4 days. The average length of stay for a patient in the US is 4.5 days. Many hospitals know that length of stay is an important metric to keep track of and that striving for shorter lengths of stay is better. Evidence suggests that the length of a patient’s stay determines their experience and outcome [1]. However, our study was not focused on prolonged hospital stay, hypothesizing that patient factors unrelated to surgical quality would also significantly impact LOS; we also evaluated the influence of these factors on complicated and uncomplicated VATS lobectomies, as reported by Giambrone [14]. For this reason, the regression analysis focused on the effects of the independent variables on hospital stay based on the presence or absence of complications. We found that the only variables not associated with LOS were FEV1 and blood loss. The advantages of minimally invasive lobectomy compared to open approaches have been extensively described [15], and in recent years, ERAS protocols have been growing interest [16]. ERAS advantages result in a shorter duration of stay in the hospital and lower costs without affecting readmission rates [17]. However, evidence for the benefit of ERAS protocols is limited, and few studies have addressed its impact on the outcome of VATS [9]. Nevertheless, the application of ERAS protocols to minimally invasive approaches represents a significant change in practice and a potential optimization in the use of resources. For this reason, identifying demographic factors, comorbidities, and surgical features associated with LOS will become critically important in defining patients’ eligibility for fast-track programs. Age is an intuitive risk factor leading to the careful consideration of physiologic age when offering a resection to an older patient. The minimally invasive approach is recommended for older patients undergoing pulmonary resections [6]. We found that the FEV1 and DLCO/VA levels were also predictive factors of LOS. Preoperative pulmonary rehabilitation for high-risk patients with chronic obstructive pulmonary disease is an effective strategy to reduce the risk of prolonged hospital stays [18]. The ESTS Minimally Invasive Thoracic Surgery Interest Group investigated significant intraoperative complications during VATS resections [19,20]. They found a conversion rate to open thoracotomy in 5.5% of cases. In our series, the overall conversion rate was 9.4%, and these patients had a prolonged LOS, supporting previous studies [21]. Regarding the surgical approach, the uniportal approach was associated with shorter LOS. However, the sample size of the uniportal VATS-L was significantly smaller than that of the three-port techniques, limiting the statistical power to identify this factor associated with the outcomes of interest. A recent meta-analysis by Harris et al. [22] compared the outcomes of multi-portal vs. uniportal VATS lobectomies for NSCLC. The results showed significant advantages of the uniportal approach regarding hospital stay, chest drain duration, and postoperative complications. Further randomized studies are needed to validate the benefits of the uniportal approach. The duration of surgery results from complex interactions between patient and disease characteristics, surgeon expertise, and system-level processes [23]. In our study, an operative time shorter than 240 min was associated with a reduced LOS. These findings support operative time as a meaningful metric in risk-adjustment methods for outcomes evaluation, performance evaluation, and comparative research. Although the correlation between hospital surgical volume and patient outcomes may appear intuitive, as previously reported [24], there is no unanimous agreement [25]. Our study identifies a clear association between hospital volume and LOS. Hospital volume appears to be an essential factor in determining LOS. This finding might be explained by the fact that higher hospital volumes may increase and preserve the experience of surgeons, thus improving the skills of the wider surgical team, including anesthetists, which could lead to a reduction in early postoperative morbidity [26]. Optimal postoperative analgesia after VATS lobectomy remains an open issue [27]. In the era of fast-track protocols, the role of thoracic epidural analgesia remains controversial [28], and a paravertebral block is effective in pain management with fewer side effects. This cohort shows that epidural placement during VATS lobectomy is associated with significantly longer LOS, and further studies are needed to confirm this finding [29].

### Limitations

This study has potential limitations. The selection bias intrinsic in retrospective extensive data analysis that includes patients operated on in different centers should be considered when evaluating the results. Furthermore, the registry’s exclusion of open lobectomies limits any comparison to conventional surgery. The Italian VATS group database 1.0 does not record the number of cases handled by each surgeon. As a result, the learning curve for minimally invasive surgery is not uniform throughout the participating institutions. These figures may not correctly reflect the influence of case volume since the caseload may be unevenly distributed within a unit. Because the Italian VATS Group is a volunteer database, our cohort, although highly representative, does not include all surgically treated NSCLC patients. Information bias, which included measurement errors and misclassifications, was possible, and missing information could result in a loss of statistical power. Our selection of the first tertile for defining reduced LOS can be viewed as an arbitrary cut-off in the absence of a predefined clinically acceptable value in the literature. As a result, this study is prone to selection bias, as the patients discharged early were a distinct subset of patients compared to the more extended LOS cohort. However, this selection bias allows us to generalize the group of patients considered safe by their physician for discharge at an earlier postoperative date in a multi-institutional sample. Additional limitations include the lack of information regarding the readmission rates and pre and postoperative care pathways, hospital characteristics, and variables outside patient characteristics (e.g., social/family environment).

## 5. Conclusions

Demographic, clinical, and surgical variables are associated with early discharge after VATS lobectomy. This study indicates that these characteristics are associated with early discharge. This knowledge can be used with clinical judgment to identify patients appropriate for fast-track protocols.

## Figures and Tables

**Table 1:** Patients’ preoperative characteristics.

Variables	Overall	LOS > 4	LOS ≤ 4	Converted
Age, mean (SD)	67.6 (9.9)	68.4 (9.7)	66.2 (10.1)	67.9 (10)
Age, median [1Q–3Q]	69 [62–75]	70 [63–75]	68 [61–73]	70 [63–75]
Sex				
Male	6121 (59.8)	4002 (62.7)	2119 (54.9)	659 (67.9)
Female	4119 (40.2)	2381 (37.3)	1738 (45.1)	309 (32.1)
LOS	5 [4–7]			7 [5–9]

**Table 2:** Patient’s preoperative comorbidities.

Variables	Overall N (%)	LOS > 4 (6383) N (%)	LOS ≤ 4 (3857) N (%)	*p*-Value
Myocardial infarction	1021 (9.9)	728 (11.4)	293 (7.6)	<0.001
Congestive heart failure	304 (3)	232 (3.6)	72 (1.9)	<0.001
Peripheral vascular disease	1579 (15.4)	1078 (16.9)	501 (13)	<0.001
Cerebrovascular disease	598 (5.8)	421 (6.6)	177 (4.6)	<0.001
Dementia	54 (0.5)	41 (0.6)	13 (0.3)	0.039
COPD	2160 (21.1)	1523 (23.7)	637 (16.5)	<0.001
Connective tissue disease	247 (2.41)	153 (2.4)	94 (2.4)	0.898
Peptic ulcer disease	386 (3.8)	226 (3.5)	160 (4.2)	0.118
Diabetes mellitus	1331 (13)	865 (13.5)	466 (12.1)	0.154
Chronic kidney disease	296 (2.9)	211 (3.3)	85 (2.2)	0.001
Malignant lymphoma	88 (0.9)	61 (1)	27 (0.7)	0.174
Solid tumor	2312 (22.6)	1473 (23.1)	839 (21.7)	0.134
Liver impairment	300 (2.9)	190 (3)	110 (2.8)	0.928
Hemiplegia	17 (0.2)	13 (0.2)	4 (0.1)	0.229
Leukemia	56 (0.5)	35 (0.6)	21 (0.5)	0.979
Aids	15 (0.1)	8 (0.1)	7 (0.2)	0.472
Neoadjuvant treatments	323 (3.1)	198 (3.1)	125 (3.2)	0.697
Charlson comorbidity index	4 [3–6]	5 [3–6]	4 [3–5]	<0.001

**Table 3:** Preoperative pulmonary function tests.

Variables	Overall Median [1Q–3Q]	LOS > 4 Median [1Q–3Q]	LOS ≤ 4 Median [1Q–3Q]	*p* Value
FEV1 value (L)	2.3 [1.9–2.8]	2.3 [1.8–2.8]	2.3 [1.9–2.8]	<0.001
FEV1%	94 [80–107]	92 [79–106]	96 [82–109]	<0.001
FVC value (L)	3.1 [2.5–3.7]	3.1 [2.5–3.7]	3.1 [2.5–3.7]	0.097
FVC (%)	100 [87–113]	99 [86–112]	101 [89–114]	<0.001
Tiffenau index	75.8 [68.7–81.9]	75.5 [67.9–81.6]	76.6 [69.9–82.5]	<0.001
DLCO/VA (%)	83 [70–96]	81 [69–94]	85 [73–98]	<0.001
ECOG	0 [0–1]	0 [0–1]	0 [0–1]	<0.001

**Table 4:** Patients’ intraoperative features.

Variables	Overall Median [1Q–3Q]	LOS > 4 Median [1Q–3Q]	LOS ≤ 4 Median [1Q–3Q]	*p*-Value
Operative time (min)	175 [135–215]	180 [141–230]	160 [125–195]	<0.001
Blood loss (mL)	100 [50–185]	100 [60–200]	100 [50–150]	<0.001
Harvested lymph nodes (n°)	11 [8–16]	11 [7–16]	12 [8–16]	0.0113

**Table 5:** Patients’ surgical findings.

Variables		Overall N (%)	LOS > 4 (6383) N (%)	LOS ≤ 4 (3857) N (%)	*p* Value
Adhesiolysis		2370 (23.1)	1605 (25.1)	765 (19.8)	<0.001
Conversion		961 (9.4)	802 (12.6)	159 (4.1)	<0.001
Surgical approach					
	Uniportal	1289 (12.6)	571 (9)	713 (18.5)	<0.001
	Anterior—Copenhagen	7061 (68.9)	4544 (71.2)	2517 (65.3)	<0.001
	Lateral—McKenna	245 (2.4)	185 (2.9)	60 (1.6)	<0.001
	Anterior—D’Amico	1420 (13.9)	920 (14.4)	500 (13)	0.038
	Totally endoscopic—Gossot	175 (1.7)	114 (1.8)	61 (1.6)	0.436
	Posterior Edinburgh—Walker	50 (0.5)	44 (0.7)	6 (0.2)	<0.001
Nodal dissection					
	Radical node dissection	6330 (61.8)	3943 (61.8)	2387 (61.9)	0.908
	Sampling node dissection	3693 (36.1)	2322 (36.4)	1371 (35.5)	0.395
	No lymph node dissection	217 (2.1)	118 (1.8)	99 (2.6)	0.014
Interlobar Fissures division (Technique)					
	Stapler	8219 (88.1)	5246 (90.6)	2973 (84)	<0.001
	Electro cautery	419 (4.5)	292 (5.1)	127 (3.6)	0.001
	Bioenergy sealer	690 (7.4)	249 (4.3)	441 (12.4)	<0.001
Type of lobectomy					
	Middle lobectomy	796 (8.1)	387 (6.3)	409 (11.3)	<0.001
	Lower lobectomy	3466 (35.4)	2175 (35.2)	1291 (35.7)	0.249
	Upper lobectomy	5350 (54.6)	3473 (56.2)	1877 (51.9)	<0.001
	Lower or upper bilobectomy	185 (1.9)	38 (1.1)	147 (2.4)	<0.001
Pathology—Benign		367 (3.6)	206 (3.2)	161 (4.2)	0.013
Pathology—Malignant	Metastasis	456 (4.5)	214 (3.3)	242 (6.3)	<0.001
Pain relief techniques					
	Intercostal block	2981 (30.9)	1803 (30.2)	1178 (32.2)	0.041
	Pericostal catheters	350 (3.6)	231 (3.9)	119 (3.3)	0.116
	Peridural catheters	2317 (24.1)	1620 (27.1)	697 (19)	<0.001
	Continuous opioids infusion	3985 (41.4)	2318 (38.8)	1667 (45.5)	<0.001
P stage					
	IA	2379 (23.2)	1475 (23.1)	904 (23.4)	0.028
	IB	2238 (21.9)	1370 (21.5)	868 (22.5)	0.003
	IIA	2495 (24.4)	1647 (25.8)	848 (22)	0.007
	IIB	523 (5.1)	354 (5.6)	169 (4.4)	0.059
	III	611 (6)	410 (6.4)	201 (5.2)	0.081
	IV	144 (1.4)	100 (1.6)	44 (1.1)	0.228
	missing	1850 (18.1)	1027 (16.1)	823 (21.3)	

**Table 6:** Patients’ postoperative events.

	Total	LOS ≤ 4	LOS > 4	*p*-Value
Atrial fibrillation	721 (7%)	88 (2.3)	633 (9.9)	<0.001
Prolonged air leak (>7 days)	830 (8.1)	25 (0.6)	805 (12.6)	<0.001
Persistent pleural space	268 (2.6)	22 (0.6)	246 (3.8)	<0.001
Pneumonia, pleural effusion, empyema	333 (3.2)	24 (0.6)	309 (4.8)	<0.001
Atelectasis	199 (1.9)	15 (0.4)	184 (2.9)	<0.001
Sputum retention	267 (2.6)	16 (0.4)	251 (3.9)	<0.001
Hemothorax	131 (1.3)	12 (0.3)	119 (1.9)	<0.001
Blood transfusion	215 (2.1)	17 (0.2)	198 (3.1)	<0.001
Acute renal failure,	56 (0.5)	0 (0)	56 (0.9)	<0.001
Diarrhea, pancreatitis, etc.	27 (0.3)	4 (0.1)	23 (0.4)	0.014
Postoperative ICU	441 (4.3)	79 (2)	362 (5.7)	<0.001
Pain day 1, median [1Q–3Q]	3 [2–4]	3 [2–3]	3 [2–4]	<0.001
Pain day 2, median [1Q–3Q]	2 [1–3]	2 [1–3]	2 [1–4]	<0.001
Pain day 3, median [1Q–3Q]	2 [1–3]	2 [1–2]	2 [1–3]	<0.001
Pain discharge day, median [1Q–3Q]	1 [1–2]	1 [1–2]	1 [1–2]	0.8103
Chest drains duration, median [1Q–3Q]	4 [3–5]	3 [2–4]	4.5 [3–6]	<0.001
Air leak duration, median [1Q–3Q]	0 [0–2]	0 [0–0]	1 [0–3]	<0.001

**Table 7:** Univariable linear regressions of variables possibly associated with a longer length of hospital stay.

Variable	*p* Value
Activity Volume (ref < 200)	0.250
Age	0.17
COPD	0.28
FEV1%	0.46
DLCO/VA%	0.98
Conversion	0.034
Operative time (min)	0.19
Blood loss (mL)	0.354
Uniportal approach	0.52

**Table 8:** Multiple logistic regression analysis of the effects of the independent variables on hospital stay based on the presence or absence of complications.

Variable		Patients without Complications			Patients with Complications		
		OR	95% CI	*p* Value	OR	95% CI	*p* Value
Activity Volume (ref < 200)	200–365	0.913	0.78–1.07	0.250	0.918	0.62–1.36	0.668
	>365	0.458	0.40–0.53	<0.001	0.566	0.40–0.81	0.002
Age	≥70	1.453	1.29–1.64	<0.001	1.661	1.24–2.22	0.001
COPD	ref.no	1.230	1.05–1.44	0.010	1.395	0.98–1.99	0.066
FEV1 (%)	≥70%	0.899	0.72–1.13	0.353	0.720	0.45–1.15	0.169
DLCO/VA (%)		0.992	0.98–0.99	<0.001	0.990	0.98–1.00	0.005
Conversion	ref. no	2.768	2.15–3.56	<0.001	1.331	0.78–2.27	0.249
Operative time (min)	≥240 min	1.510	1.25–1.83	<0.001	1.252	0.84–1.86	0.266
Blood loss (mL)	≥600 mL	3.922	1.13–13.60	0.031	1.266	0.42–3.85	0.678
Uniportal approach	ref.no	0.504	0.41–0.61	<0.001	0.260	0.18–0.37	<0.001
Bioenergy sealer use	ref.no	0.478	0.38–0.60	<0.001	0.576	0.34–0.98	0.043
Intercostal block	ref.no	0.744	0.66–0.84	<0.001	1.233	0.89–1.70	0.201

## Data Availability

The data underlying this article will be shared upon reasonable request to the corresponding author.

## Supplementary Materials

The following supporting information can be downloaded at: https://www.mdpi.com/article/10.3390/jcm11247356/s1. Reference [30] is cited in the supplementary materials.
